# Characterization of Active Edible Films based on Citral Essential Oil, Alginate and Pectin

**DOI:** 10.3390/ma11101980

**Published:** 2018-10-15

**Authors:** Valentina Siracusa, Santina Romani, Matteo Gigli, Cinzia Mannozzi, Juan Pablo Cecchini, Urszula Tylewicz, Nadia Lotti

**Affiliations:** 1 Department of Chemical Science, University of Catania, Viale A. Doria 6, 95125 Catania (CT), Italy; 2 Department of Agricultural and Food Sciences-DISTAL, Campus of Food Science, University of Bologna, P.zza Goidanich 60, 47521 Cesena, Italy; santina.romani2@unibo.it (S.R.); cinzia.mannozzi2@unibo.it (C.M.); urszula.tylewicz@unibo.it (U.T.); 3 Department of Chemical Science and Technologies, University of Rome Tor Vergata, Via della Ricerca Scientifica 1, 00133 Rome, Italy; matteo.gigli@unibo.it; 4 Universidad Nacional de Rafaela, Bv. Roca 989, Rafaela, 3000 Santa Fe, Argentina; jpcecchini@gmail.com; 5 Department of Civil, Chemical, Environmental and Materials Engineering, University of Bologna, via Terracini 28, 40131 Bologna, Italy; nadia.lotti@unibo.it

**Keywords:** edible film, alginate film, pectin film, essential oil, barrier properties, mechanical properties

## Abstract

Thermal, structural and physico-chemical properties of different composite edible films based on alginate and pectin with the addition of citral essential oil (citral EO) as an agent to improve barrier properties, were investigated. The obtained films were clear and transparent, with a yellow hue that increased with citral EO addition. All the films displayed good thermal stability up to 160 °C, with a slight improvement observed by increasing the amount of citral EO in the composites. Gas transmission rate (GTR) strongly depended on the polymer structure, gas type and temperature, with improvement in barrier performance for composite samples. Also, citral EO did not exert any weakening action on the tensile behavior. On the contrary, an increase of the elastic modulus and of the tensile strength was observed. Lastly, water contact angle measurements demonstrated the dependence of the film wettability on the content of citral EO.

## 1. Introduction

Edible coatings and films belong to an environmental-friendly technology aimed at enhancing food safety, quality and handling properties by creating a biodegradable semi-permeable barrier protection to water vapor, oxygen and carbon dioxide coming from the external environment. Edible coating is a thin edible substance layer, applied in a liquid form directly on the surface of different food products, mainly fruits and vegetables [1,2,3,4,5]. Edible films are a thin layer obtained as a solid sheet, which can be applied as a wrapping on the food product. For fresh fruit and vegetables, the creation of a wrong moisture and gas atmosphere may lead to weight loss and respiration rate reductions, with a consequent acceleration of the senescence process and a worsening of the visual gloss of coated commodities. Several studies have identified the necessity to evaluate mechanical (flexibility, tension), thermal, optical (brightness, opacity), wettability and morphological properties of edible films, as it creates a modified atmosphere that influences the gas transfer and further becomes a barrier for aromatic compound transferring [6,7]. The above-mentioned characteristics depend on several parameters related to the coating and film composition, such as preparation conditions (solvent, pH, components concentration, temperature) and type of added additives (cross-linking agents, antimicrobials, plasticizers, emulsifiers).

Recently, further potential applications were studied in order to formulate edible coatings and films with special functionalities [8]. They could be used as carriers of antioxidants, flavoring and/or coloring agents and antimicrobials to improve food safety and quality [9,10].

Edible coating and film formulations require several components. At least one of them needs to have structural properties. Between the different compounds that could be used in the formulation, alginate and pectin have been widely investigated [1]. They are hydrocolloid compounds coming from respectively seaweed extracts (brown algae) and plant tissue. Both belong to the polyuranoates group and are natural ionic polysaccharides giving rise to a chain–chain association and forming hydrogels. In order to design new films with improved and selective mechanical and barrier properties, it is important to understand their compatibility [1,11,12]. Further, essential oils represent a very interesting ingredient for edible film formulation with the purpose of realizing active and natural packaging materials [13,14]. Due to their natural origin and special functionality (antioxidant/antimicrobial) they could be selected to extend food shelf-life and add value to the product. However, the introduction of edible films for food packaging application may affect several properties such as optical, tensile, permeability and so on [15]. This, in turn, could influence the consumer acceptability. Citral EO was selected since it has been proved to have a positive effect against food spoilage microorganisms, as described by Guerreiro et al. [16].

The objective of this study was to investigate the final properties of edible films based on sodium alginate (SA) and pectin (Pe), with the addition of citral essential oil (citral EO).

The optimization of an edible film composition is one of the most important steps [17,18], as it must be formulated in order to maintain or increase the quality of fruit and vegetables. In this framework, physico-chemical and structural analyses have been performed on the prepared formulations to evaluate the properties of interest for the in-use application.

## 2. Materials and Methods

### 2.1. Materials

Citral essential oil, sodium alginate, glycerol (≥99.5%), Tween^®^20 and Pectin from citrus peel (Galacturonic acid ≥74.0%) were purchased all from Sigma-Aldrich (Darmstadt, Germany, Europe) and used as received.

### 2.2. Film Preparation

Edible films were prepared using the solution casting method. Three different compositions were tested: 2% (w/w) of SA, 2% (w/w) of Pe and a combination of 1% (w/w) sodium alginate and 1% (w/w) pectin (SA + Pe). 1.5% (w/w) of glycerol and 0.2% (w/w) of Tween^®^ 20 were added to each solution, which was subsequently dissolved in distilled water. Moreover, different amounts (0, 0.15, 0.3% w/w) of citral essential oil (citral EO) as antimicrobial agent were introduced in the formulation and homogeneously dispersed. In total, nine different films were obtained, as reported in Table 1.

All solutions were heated at 70 °C for 30 min under conditions of stirring (until complete dissolution) and homogenized at 5000 rpm for 2 min. Afterwards, about 27 g of solution was used to obtain 1 g of soluble solids in petri dishes (ø 90 mm). Air bubbles were removed by placing the film forming solution under vacuum (60 mbar, 15 min). Films were dried at 40 °C for 5 h to ensure a complete solvent removal, and then stored at room temperature in a desiccator before characterization.

### 2.3. Film Thickness and Gas Barrier Properties

Film thickness was measured by using a Sample Thickness Tester DM-G, consisting of a digital indicator (MarCator 1086 type, Mahr GmbH, Esslingen, Germany) connected to a PC. The reading was made twice per second measuring a minimum, a maximum and an average value, with a resolution of 0.001 mm. The reported results represent the mean thickness value of three experimental tests run in 10 different positions of each film at room temperature.

The determination of the gas barrier properties was performed by a manometric method using a Permeance Testing Device, type GDP-C (Brugger Feinmechanik GmbH, Munchen, Germany), in accordance with the ASTM 1434, DIN 53536, ISO 15105-1 and with the Gas Permeability Testing Manual-2008. The equipment consisted of an upper and a lower chamber, as previously described [19]. The film sample, of approximately 3 cm × 3 cm in size, was placed between the two chambers. A film mask was used to cover the remaining section of the permeation chamber. The top chamber was filled with the dry test gases at ambient pressure. The gas permeation was determined by evaluating the pressure increase in the bottom chamber, which had been previously evacuated. Ambient temperature fluctuations during the test were controlled by an automatic temperature compensation software, which minimizes gas transmission rate (GTR) temperature deviations. The following conditions were adopted: gas stream of 100 cm^3^/min, pure food grade gases with 0% RH, active sample area of 0.785 cm^2^. *Method A* (with evacuation of top and bottom permeation chamber) was employed in the analysis, as reported in the Gas Permeability Testing Manual (2008).

In order to determine the activation energies of the permeation process, the films were tested at three different temperatures (8, 15 and 23 °C), in the same operating conditions as reported above. Chamber and sample temperature were kept constant by an external thermostat HAAKE-Circulator DC10-K15 type (Thermo Fischer Scientific, Waltham, MA, USA). All experiments were done in triplicate and the results are presented as mean values.

### 2.4. Mechanical Properties

Tensile testing was performed by using a Zwick Roell Texture machine mod. Z2.5 (Ulm, Germany), equipped with a rubber grip and a 500 N load cell. A pre-load of 1MPa with a pre-load speed of 5 mm/min was applied. Stress-strain measurements were performed on rectangular films (5 mm width and 50 mm length) with an initial grip separation of 23 mm and a crosshead speed of 5 mm/min. Five different specimens was analyzed for each film and the results are provided as the average value ± standard deviation. All measurements have been carried out in accordance with ASTM D882-09.

### 2.5. Thermo-Gravimetric Analysis (TGA)

Thermo-Gravimetric Analysis (TGA) was carried out under nitrogen atmosphere by means of a Perkin Elmer TGA7 apparatus (Perkin Elmer, Waltham, MA, USA). Gas flow of 30 mL/min and heating scan of 10 °C/min, over a temperature range 40–800 °C, were used for the analyses. Samples mass of 10 mg were used for the experiments.

### 2.6. Attenuated Total Reflectance Infrared Spectroscopy (ATR-IR) Analysis

Experimental ATR modified absorbance spectra were collected on a Perkin Elmer FTIR (Perkin Elmer 1725× Spectrophotometer, Labexchange Gropu, Burlandingen, Germany) over the range 650–4000 cm^−1^, with a resolution of 4.0 cm^−1^. The results are presented as an average of 10 experimental tests, run on 10 different samples point. 64 scans were recorded on each sample. The experiments were performed at room temperature, without any preliminary samples’ treatments.

### 2.7. Water Contact Angle Determination (WCA)

Static contact angle measurements were performed on edible films using a KSV CAM101 (KSV Instrument, Elsinki, Finland) instrument at ambient conditions, by recording the side profiles of deionized water drops (4 µL) deposited on the film surface. The measurement was taken 2 s after the drop deposition to ensure its stabilization, yet to minimize water absorption and evaporation. At least five drops were observed on different areas for each film (both on the top and on the bottom side), and contact angles were reported as the average value ± standard deviation.

### 2.8. Color

The color of film samples was measured using a HunterLab ColorFlex EZ 45/0° color spectro-photometer (Hunterlab, Reston, VA, USA), with D65 illuminant, 10° observer, according to ASTM E308. The measurements were made using CIE Lab scale. The instrument was calibrated with a black and white tile (L* 93.47, a* 0.83, b* 1.33) before the measurements. Results are expressed as L* (luminosity), a* (red/green) and b*(yellow/blue) parameters.

The total color difference (ΔE) was calculated using the following equation:

ΔE = [(L* − L’)^2^ + (a* − a’)^2^ + (b* − b’)^2^]^1/2^ (1)

where L*, a* and b* are the color parameter values of the sample and L’, a’ and b’ are the color parameter values of the standard white plate used as the film background (L’ = 66.39, a’ = −0.74, b’ = 1.25). The analyses were conducted in five repetitions. A mean value recorded for the top side and bottom side is reported.

### 2.9. Morphology Evaluation

The films’ morphology was determined by using a Nikon upright microscope (Eclipse Ti-U, Nikon Co, Shanghai, Japan) with a standard light. Samples were observed in black and white and the images were recorded at a 20× magnification.

### 2.10. Statistical Analysis

Significance of the different edible films effects was evaluated by means of one-way analysis of variance (ANOVA, 95% significance level) using the software STATISTICA 6.0 (Statsoft Inc., Tulsa, OK, USA).

## 3. Results and Discussions

In general, all films appeared homogeneous and transparent, without evident pores and crakes. It can be noticed that SA-based films were visually more transparent than Pe-based ones. Similar observations were made by Galus and Lenart [11] in their study on edible films based on sodium alginate and pectin. Visually, the addition of citral essential oil added a bright yellow color to the prepared films.

### 3.1. Thickness and Gas Barrier Properties

#### 3.1.1. Thickness

Films thickness ranged from 94 to 127 μm as reported in Table 2, showing a slight variation of thickness within the different formulations, associated to the film casting technique. This is a crucial parameter for the calculation of mechanical and barrier properties. It depends on the film preparation method, on the flatness of the dish surface and on the film formation during the drying process. Despite the same volume of film forming solutions were used for the film casting, the drying time for complete solvent evaporation increased with the citral EO concentration (anyway remaining in all cases within 5 h). The homogeneity of the films increased with the solvent evaporation time. Consequently, as reported in literature, the thickness tent to be lower in a well-organized and dense network [20]. Differences in the film structure may be attributed to the effect on the drying kinetics of the liquid film-forming dispersion thickness [21]. Several authors reported the effects of thickness on the permeability of edible matrix [22,23].

The thickness values have been used to calculate gas permeability and tensile properties.

#### 3.1.2. Gas Barrier Properties

As it is well known, oxygen might cause food oxidation, which in turn influences various food properties such as odour, colour, flavour and nutrient content [24,25]. The film ability to retard oxidation or degradation of the product is an important characteristic that affects the final product quality and food shelf-life. Therefore, gas barrier properties should be taken into due account. Furthermore, the incorporation of other compounds, such as essential oils, into the polymer matrix could contribute to the modification of their barrier behavior.

Gas transmission rate (GTR) strongly depends on the polymer structure, gas type and temperature. Edible films barrier properties were examined at three different temperatures [26,27]: 8 °C (home and supermarket refrigerated storage temperature), 15 °C (abusing temperature) and 23 °C (room temperature). As reported by Marklinder and Eriksson [27], a typical recommended storage temperature for perishable food items should not exceed 8 °C, with the exception for minced meat that should be stored at 4 °C maximum. Further, in almost all the refrigerators the temperature ranges between 5–8 °C, but it is not uncommon that in certain zones of the refrigerators the temperature increases to values higher than 12 °C.

Data recorded with 100% pure food grade O_2_ and CO_2_ are reported in Table 2.

The matrix composition influenced the diffusion of the gas molecules through the polymer, resulting in a substantial variation of the gas transmission. Citral EO caused a modification of the barrier performances, correlated to the compatibility between alginate and pectin, ultimately resulting in effective gas molecule permeation through the films. The impact of lipid addition on the microstructure of the coating films is a determining factor in barrier efficiency. The microstructure of the films is affected by the physical state of the essential oil and its distribution in the polymer matrix. The liquid state of citral EO could favor molecular mobility, promoting the transport of gas molecules through the films. In turn, its distribution is another important factor. The more homogeneous is the distribution, the lower is the GTR. Antarés and Chiralt [28] reported that the addition of essential oil causes an increase of the oxygen permeability due to its hydrophobic character. However, a different trend can be described for the materials object of the present study. Indeed, a GTR decrease, particularly evident at lower temperatures, has been observed. The data here presented are well in accordance with the results reported by Rojas-Graü and coworkers [8]. They observed that the introduction of antimicrobials agents (oregano, carvacrol and lemongrass oil) did not affect the oxygen permeability of the films, while a slight decrease was observed by adding citral EO to alginate-based edible films.

It must be considered that the film barrier behavior at 8 °C is the most crucial for food packaging applications, as it corresponds to home and commercial (supermarket) storage conditions. At this temperature the gas transmission rate of O_2_ and CO_2_ is very similar: CO_2_ permeates maximum 1.4 times faster than O_2_, as it can be seen from the perm-selectivity ratio reported in Table 2.

As reported in the literature [29], for non-condensable gases the perm-selectivity ratio is relatively constant and is not correlated to the polymer chemical structure. Moreover, CO_2_ transmission across the matrix is in general about six times faster than O_2_ ones.

Furthermore, the perm-selectivity ratio changes with the temperature, i.e., it usually increases as the temperature decreases. On the contrary, our study evidenced a different trend, as the permselectivity remained almost constant in all cases.

**Figure 1:**
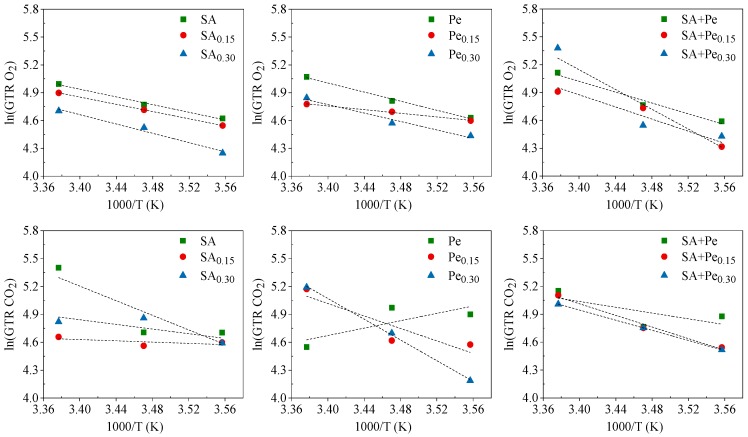
Arrhenius plot of O_2_ and CO_2_ GTR coefficient for SA, Pe and SA + Pe films.

The Arrhenius model was used to describe the permeation dependence on the temperature and to calculate the activation energy of the gas transmission (E_GTR_) process. In Figure 1, natural logarithm (ln) of GTR has been plotted as a function of the reciprocal of the absolute temperature (1/T).

Data reported in Table 2 well fit the theoretical relation (high R^2^ value) in most cases, indicating a good correlation between permeability and temperature for both CO_2_ and O_2_. As expected, the higher the temperature, the higher the permeability. In addition, E_GTR_ reflected the dependence of the barrier behavior from the temperature for each polymer matrix. The low activation energy demonstrated a low dependence of the gas transmission process on the temperature.

In conclusion, the GTR data here reported confirm the antioxidant action of citral EO, which is due to the promotion of oxygen barrier capacity, as already reported by Rojas-Graü et al. and Shahbazi [8,9,10,11,12,13,14,15,16,17,18,19,20,21,22,23,24,25,26,27,28,29,30]. The samples containing citral EO showed an interesting improvement of the gas barrier behavior, making them suitable as edible films for food more susceptible to oxidative degradation. As can be evicted from the data reported, the GTR value decreased when citral EO was added. The barrier properties improvement is responsible of the feasibility of formed films to meet the desired functionality for the product to be packed, as was reported also by Avila-Sosa et al. [31] in their study of chitosan and oregano essential oil, as well as their combination, for antimicrobial food packaging application.

### 3.2. Mechanical Properties

The tensile behavior of the films under study is reported in Table 3. Films were analyzed in terms of their elastic modulus (E), stress at yield (σ_y_) and at break (σ_b_), and elongation at yield (ε_y_) and at break (ε_b_). Generally speaking, the tensile properties of edible films and coatings are dependent on film constituents, relative proportion and preparation conditions [28].

Neat SA and Pe films showed very comparable tensile properties, although the elongation at break of SA was higher, while SA + Pe films displayed the lowest elastic modulus and stress at break and the highest elongation at break among the studied samples. The addition of citral EO caused a significant change of the mechanical properties of all the formulations. A general increase of E, σ_y_ and σ_b_ can be observed, while no significant differences can be noticed for ε_b_ (Table 3). Also, the introduction of different amounts of citral EO did not influence the tensile behavior of the samples. In all the investigated samples, the yield phenomenon is visible.

The effect of the essential oil addition on the mechanical properties of edible films is quite complex and conflicting results have been reported in the literature, since both enhancement and weakening of the tensile characteristics have been observed [28]. Specific interactions between the film constituents and the oil, such as crosslinks, different structural arrangements of the components or the formation of heterogeneous biphasic structures, must be taken into consideration [28].

Our study demonstrates that citral EO does not have any detrimental effect on the tensile properties of SA and Pe based formulations. On the contrary, an enhancement of the tensile strength and of the film rigidity has been obtained. The results indicate that not only the film microstructure was preserved, yet citral EO played a reinforcement role. Also, the introduction of a hydrophobic compound may have caused a reduction of the film water absorption, this latter acting as plasticizer and causing a decrease of E and σ_b_ in the neat films as compared to the composites.

In conclusion, the composite films, in particular those containing a higher amount of citral EO, displayed improved mechanical resistance with respect to the neat films, thus making them more suitable for food wrapping applications.

### 3.3. Thermogravimetric Analysis

Table 4 reports the thermogravimetric data of the materials under study, i.e., T_onset_ (the temperature at which the degradation starts), T_max_ (the temperature of the maximum degradation rate), and m_res,600 °C_ (the residual mass at 600 °C).

The degradation patterns of the different films are very similar, although a slight improvement of the stability can be seen by increasing the amount of citral EO in the composites (Table 4).

**Figure 2:**
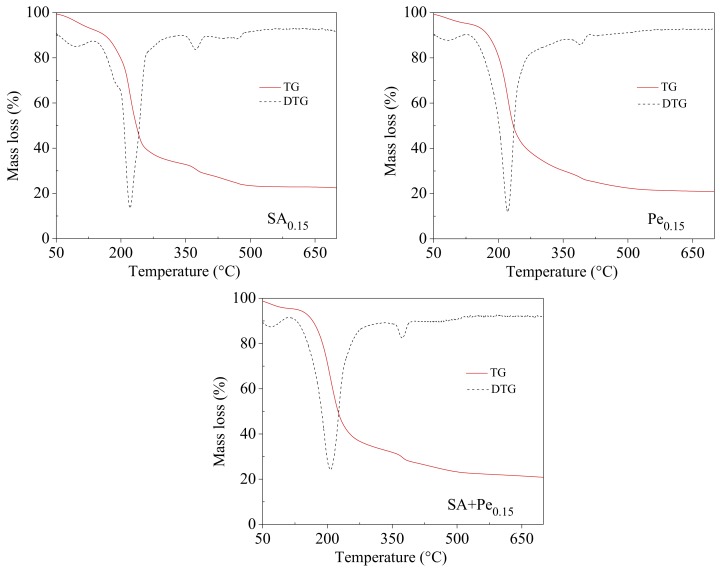
Thermogravimetric curves of SA_0.15_, Pe_0.15_ and SA + Pe_0.15_ under N_2_ flow.

Figure 2 reports the thermogravimetric curves of SA_0.15_, Pe_0.15_ and SA + Pe_0.15_, together with their derivative.

Two main degradation steps can be outlined (Figure 2). The first one, which takes place below 160 °C, is due to film dehydration. The second step, which comprises two distinct peaks (as evidenced by the DTG curves), is related to the decomposition of the film components. In particular, the most evident mass loss (which reached the maximum in the range 210–220 °C), can be ascribed to the degradation of the polymer backbone.

### 3.4. Water Contact Angle (WCA) Measurements

**Figure 3:**
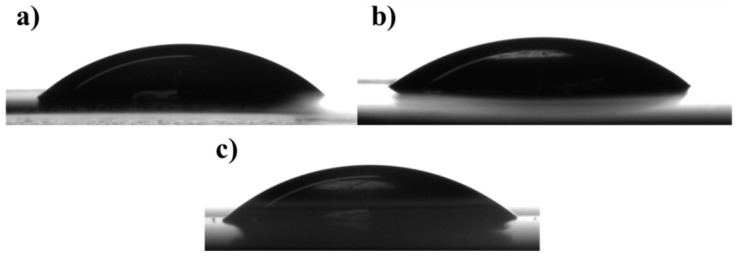
Water drops deposited on the film surface of: (**a**) SA_0.15_; (**b**) Pe_0.15_; (**c**) SA + Pe_0.15_.

Surface hydrophobicity is an important parameter to control the sensitivity of the films to water or moisture and it is usually evaluated by measuring the contact angle that forms between the film surface and a water droplet [20,32]. Generally, higher contact angle values correspond to higher surface hydrophobicity, with higher surface water repellent ability. Figure 3 collects, as an example, the images of the water drops deposited on the film surface of SA_0.15_, Pe_0.15_ and SA + Pe_0.15_. Water contact angle measurements (Table 4) demonstrated the high wettability of the prepared films, due to the presence of a high amount of ether bonds and free hydroxyl-groups. In fact, WCAs were in all cases below 50°. The addition of citral EO caused a rise of the contact angle. In particular, the higher the content of citral EO, the higher the WCA, because of the hydrophobic nature of this latter Therefore, the presence of citral EO plays a beneficial role in decreasing the wettability of the edible film, thus improving its protective action towards the wrapped food.

### 3.5. ATR-IR Analysis

The IR pattern of SA and Pe, as well as those of the composites, displayed some common and characteristic peaks (Figure S1). In particular, a broad and intense peak in the range 3270–3300 cm^−1^ due to the stretching of the OH groups, a smaller peak of the CH stretching at 2920–2930 cm^−1^, and an intense and sharp peak at 1015–1025 cm^−1^ due to the C-O-C stretching of the glycosidic bonds linking two galacturonic sugar units were observed [33,34,35].

Some differences between the SA and Pe profile can also be highlighted. Indeed, the SA spectrum reveals the presence of two intense peaks at 1602 cm^−1^ and 1408 cm^−1^, respectively due to the COO- asymmetric and symmetric stretching vibration, and with a weak shoulder at 1298 due to the CO stretching. On the contrary, Pe showed its characteristic band at 1743 cm^−1^, assigned to the stretching of the methyl esterified carboxyl groups [36]. The peak at 1607 cm^−1^ (due to the COO- asymmetric stretching) was also clearly visible. The IR pattern of the composites presented the peaks of both SA and Pe.

The identification of the characteristics peaks allowed to confirm for all films the expected structure.

### 3.6. Color

In Table 5, L*, a*, b*, and total color difference (ΔE) values were reported for each sample.

All the films were transparent. Since the color of edible films may affect the consumer acceptance, it is of primary importance that its transparency is preserved or that at least they display a color as close as possible to the natural pigment of foods on which the film is going to be applied [37]. SA based films showed slightly higher values of L* (luminosity) as compared with those composed of Pe and SA + Pe, in agreement with Galus and Lenart [11]. The addition of citral EO led to a general enhancement of L*. In particular, the increment was of the same entity in the SA samples containing different amounts of citral EO, while for Pe and SA + Pe films the L* values gradually increased with the increment of citral EO content. In terms of a* (red/green index) and b* (yellow/blue index) parameters, the samples containing Pe (both pure or in combination with SA) tended to a more yellowish color. In this case, 0.15 w/w of citral EO significantly enhanced (*p* < 0.05) the b* values. The obtained ΔE data ranged from 8 to 15. Pe-based samples showed the highest value, indicating a greater color variation, in agreement with the results reported by Galus and Lenart [11]. Samples displaying higher ΔE values (Pe, Pe_0.15_, SA + Pe and SA + Pe_0.15_) appeared also less transparent.

By citral EO addition, only a slight yellowish hue was recorded, making all those films suitable for food application.

### 3.7. Microstructure

As reported by Antarés and Chiralt [28], while conventional plastics are non-polar materials, edible films are usually hydrophilic. The incorporation of essential oil in the film forming dispersion is usually carried out by emulsification or homogenization of the aqueous solution containing the polymer. When the film is dried, droplets of lipid remain embedded into the polymer matrix, as observed by microscopy. The drying time plays an important role in determining the arrangement of the components during the film-forming step, thus the final microstructure of the edible films.

**Figure 4:**
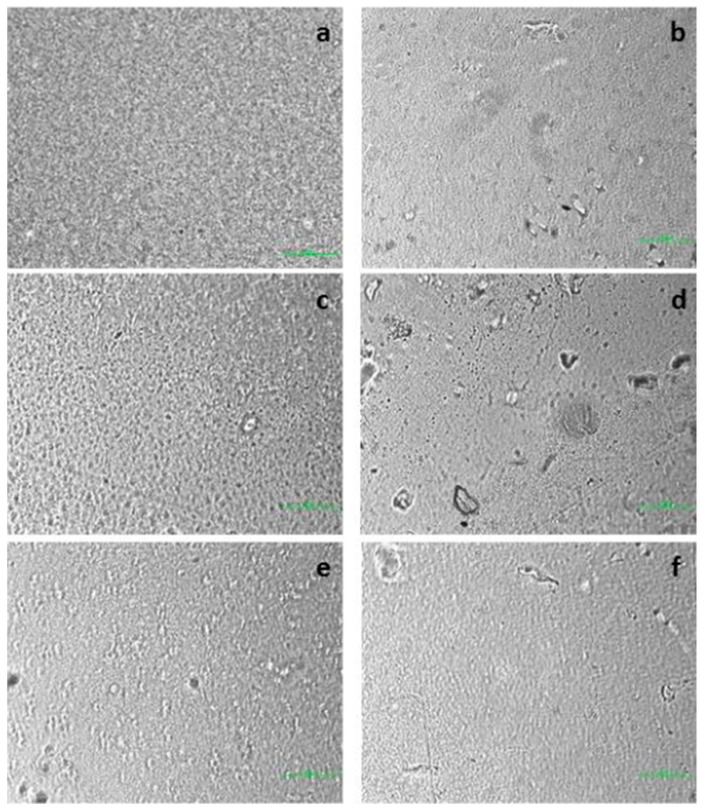
Surface micrographs of: (**a**) SA; (**b**) SA_0.30_; (**c**) Pe; (**d**) Pe_0.30_; (**e**) SA + Pe and (**f**) SA + Pe_0.30_ films.

Morphological analysis of the films under study was carried out by optical microscopy (20× magnification), to evaluate the homogeneity and the structure of the prepared films. Figure 4 shows the surface micrographs of SA (a), Pe (c) and SA + Pe (e) films and of SA_0.30_, Pe_0.30_ and SA + Pe_0.30_ (respectively (b), (d) and (f)).

SA films presented a more homogenous and uniform structure than Pe ones, in accordance with Bierhalz et al. [38], who performed the microstructural analysis on alginate and pectin based films with the addition of natamycin as antimicrobial agent. The addition of citral EO caused a mild change in the structure of all films. Furthermore, some agglomerates can be noticed, possibly indicating a non-uniform distribution of citral EO. The most irregular structure was observed in Pe-based films. The morphology of the films supported the tensile results, evidencing that a different structural arrangement of the components in the film forming dispersion significantly influences both mechanical and gas barrier properties.

## 4. Conclusions

Citral EO has been incorporated into edible films through emulsification. Film structural, physical, mechanical and barrier properties were evaluated. The combination of citral EO with SA and/or Pe gives continuous and transparent edible films. Samples with citral EO addition showed improved performances with respect to the neat ones. As compared to neat films, no substantial differences were observed in the visual appearance of the composite samples, with the exception of a slight yellowish hue. On the other hand, citral EO addition caused a different microstructure arrangement and some agglomerates. However, the film structure was not weakened as testified by the gas barrier and mechanical tests, rather a reinforcement role was exerted. Furthermore, by increasing the amount of citral EO, a slight increase of the thermal stability as well as of the surface hydrophobicity was recorded.

With the aim to extend the shelf-life of food, the addition of essential oils can provide edible films with antimicrobial properties, depending both on their nature and interaction with the polymer matrix. Further studies are necessary to optimize and verify the suitability of these natural films on food products. The oil type and its interaction with the matrix determine the effectiveness of the edible films as food packaging material. In order to understand the feasibility of edible films to improve the shelf-life of food products susceptible to oxidation or to microbial spoilage, a real food systems needs to be investigated, together with specific tests on antioxidant activity [39,40].

## Figures and Tables

**Table 1:** Concentration (w/w %) of the components in the film forming solutions.

Sample	Sodium Alginate	Pectin	Glycerol	Tween^®^ 20	Citral EO
SA	2	-	1.5	0.2	-
SA_0.15_	2	-	1.5	0.2	0.15
SA_0.3_	2	-	1.5	0.2	0.3
Pe	-	2	1.5	0.2	-
Pe_0.15_	-	2	1.5	0.2	0.15
Pe_0.3_	-	2	1.5	0.2	0.3
SA + Pe	1	1	1.5	0.2	-
SA + Pe_0.15_	1	1	1.5	0.2	0.15
SA + Pe_0.3_	1	1	1.5	0.2	0.3

**Table 2:** Film thickness, O_2_ and CO_2_ gas transmission rate (GTR) at 8, 15 and 23 °C, with the corresponding perm-selectivity ratio and GTR Activation Energy.

Sample	Thickness (μm)	O_2_-GTR (cm^3^/m^2^ d bar)			CO_2_-GTR (cm^3^/m^2^ d bar)			CO_2_/O_2_	CO_2_/O_2_	CO_2_/O_2_	CO_2_ E_GTR_ (J/mol K)	O_2_ E_GTR_ (J/mol K)
		8 °C	15 °C	23 °C	8 °C	15 °C	23 °C	8 °C	15 °C	23 °C		
**SA**	127 ± 13 ^a^	102 ± 1 ^a^	118 ± 1 ^b^	148 ± 2 ^c^	110 ± 0 ^c^	111 ± 1 ^d^	222 ± 1 ^a^	1.1	0.9	1.5	33 (0.8)	17 (1)
**SA_0.15_**	111 ± 6 ^b^	94 ± 1 ^c^	112 ± 2 ^de^	134 ± 1 ^d^	99 ± 2 ^d^	96 ± 1 ^f^	105 ± 1 ^i^	1.1	0.7	0.8	3 (0.4)	16 (1)
**SA_0.30_**	110 ± 4 ^b^	70 ± 1 ^f^	92 ± 1 ^f^	110 ± 0 ^g^	99 ± 1 ^d^	129 ± 1 ^b^	124 ± 2 ^h^	1.4	1.4	1.1	10 (0.6)	21 (1)
**Pe**	110 ± 5 ^b^	102 ± 1 ^a^	123 ± 1 ^a^	159 ± 1 ^b^	134 ± 2 ^a^	144 ± 2 ^a^	195 ± 1 ^b^	1.3	1.2	1.2	−16 (0.6)	20 (1)
**Pe_0.15_**	107 ± 1 ^bc^	99 ± 2 ^ab^	109 ± 1 ^e^	119 ± 1 ^f^	97 ±1 ^d^	101 ± 1 ^e^	176 ± 1 ^d^	1.0	0.9	1.6	28 (0.8)	8 (1)
**Pe_0.30_**	94 ± 9 ^c^	84 ± 2 ^d^	117 ± 1 ^bc^	127 ± 0 ^e^	66 ± 2 ^g^	110 ± 1 ^d^	180 ± 1 ^c^	0.8	0.9	1.4	46 (1)	19 (1)
**SA + Pe**	101 ± 3 ^bc^	98 ± 1 ^b^	117 ± 1 ^bc^	166 ± 1 ^a^	131 ± 1 ^b^	117 ± 1 ^c^	173 ± 1 ^e^	1.3	1.0	1.0	13 (0.5)	24 (1)
**SA + Pe_0.15_**	104 ± 1 ^bc^	75 ± 1 ^e^	114 ± 4 ^cd^	136 ± 1 ^d^	94 ± 1 ^e^	116 ± 2 ^c^	165 ± 1 ^f^	1.3	1.0	1.2	26 (1)	27 (0.9)
**SA + Pe_0.30_**	104 ± 5 ^bc^	84 ± 1 ^d^	94 ± 3 ^f^	117 ± 1 ^e^	92 ± 1 ^f^	116±1 ^c^	150±1 ^g^	1.1	1.2	1.3	23 (1)	44 (0.9)

Values with different letters within the same column differ significantly at *p* < 0.05 levels. Values in brackets indicate the R^2^ parameter of the fitting curve. Samples with *a* present the highest value, while samples with *d* the lowest.

**Table 3:** Mechanical data of SA, Pe and SA + Pe films.

Sample	E (MPa)	σ_y_ (MPa)	ε_y_ (%)	σ_b_ (MPa)	ε_b_ (%)
SA	526 ± 52 ^c^	14 ± 1 ^d^	4 ± 1 ^b^	25 ± 3 ^cd^	30 ± 1 ^b^
SA_0.15_	1102 ± 68 ^ab^	24 ± 2 ^b^	3 ± 1^b^	44 ± 7 ^ab^	24 ± 5 ^cd^
SA_0.30_	955 ± 74 ^b^	17 ± 5 ^c^	5 ± 2 ^b^	31 ± 11 ^c^	30 ± 3 ^b^
Pe	492 ± 51 ^c^	13 ± 1 ^de^	4 ± 1 ^b^	21 ± 2 ^cd^	18 ± 2 ^e^
Pe_0.15_	1120 ± 112 ^ab^	21 ± 2 ^bc^	3 ± 1 ^b^	42 ± 5 ^b^	20 ± 1 ^de^
Pe_0.30_	1338 ± 115 ^a^	32 ± 5 ^a^	4 ± 1 ^b^	54 ± 7 ^a^	20 ± 2 ^de^
Pe + SA	173 ± 17 ^d^	11 ± 1 ^e^	12 ± 1 ^a^	18 ± 1 ^d^	50 ± 1 ^a^
Pe + SA_0.15_	915 ± 109 ^b^	19 ± 3 ^c^	4 ± 1 ^b^	31 ± 4 ^c^	18 ± 2 ^e^
PE + SA_0.30_	1171 ± 98 ^ab^	28 ± 3 ^a^	4 ± 1 ^b^	50 ± 6 ^ab^	26 ± 2 ^bc^

Values with different letters within the same column differ significantly at *p* < 0.05 levels. Samples with *a* present the highest value, while samples with *d* the lowest.

**Table 4:** Thermogravimetric and wettability data of Sa, Pe and SA + Pe films.

Sample	T_onset_ (°C)	T_max_ (°C)	m_res,600 °C_ (%)	WCA (°)
SA	163 ± 1 ^e^	220 ± 1 ^b^	20 ± 1 ^de^	44 ± 3 ^b^
SA_0.15_	172 ± 1 ^c^	221 ± 1 ^ab^	23 ± 0 ^b^	45 ± 3 ^ab^
SA_0.30_	178 ± 1 ^ab^	221 ± 0 ^ab^	24 ± 0 ^a^	49 ± 4 ^a^
Pe	171 ± 1 ^c^	218 ± 1 ^c^	17 ± 1 ^f^	35 ± 4 ^d^
Pe_0.15_	176 ± 3 ^b^	222 ± 2 ^ab^	21 ± 1 ^cd^	38 ± 4 ^cd^
Pe_0.30_	179 ± 2 ^a^	222 ± 1 ^a^	20 ± 1 ^e^	42 ± 2 ^bc^
SA + Pe	163 ± 1 ^e^	210 ± 1 ^d^	18 ± 1 ^f^	43 ± 3 ^bc^
SA + Pe_0.15_	166 ± 2 ^d^	207 ± 1 ^e^	22 ± 0 ^bc^	46 ± 2 ^ab^
SA + Pe_0.30_	173 ± 2 ^c^	213 ± 2 ^c^	20 ± 1 ^de^	48 ± 1 ^ab^

Values with different letters within the same column differ significantly at *p* < 0.05 levels. Samples with *a* present the highest value, while samples with *d* the lowest.

**Table 5:** L*, a*, b* and total color difference (ΔE) of SA, Pe and SA + Pe films.

Sample	L*	a*	b*	ΔE
SA	86 ± 2 ^b^	−1.56 ± 0.06 ^de^	5.1 ± 0.4 ^e^	9 ± 2 ^c^
SA_0.15_	87.4 ± 0.7 ^a^	−1.69 ± 0.02 ^efg^	6 ± 1 ^de^	8 ± 1 ^c^
SA_0.30_	87.1 ± 0.3 ^a^	−1.75 ± 0.02 ^fg^	6.9 ± 0.6 ^d^	8.5 ± 0.6 ^c^
Pe	83.7 ± 0.7 ^c^	−0.17 ± 0.03 ^a^	10 ± 1 ^b^	13 ± 1 ^ab^
Pe_0.15_	84.3 ± 1.3 ^c^	−1.2 ± 0.2 ^b^	13 ± 1 ^a^	15 ± 2 ^a^
Pe_0.30_	87.9 ± 0.4 ^a^	−1.62 ± 0.08 ^def^	8 ± 2 ^cd^	8 ± 2 ^c^
SA + Pe	83.7 ± 0.8 ^c^	−1.40 ± 0.04 ^c^	7.9 ± 0.6 ^cd^	11.8 ± 0.7 ^b^
SA + Pe_0.15_	85 ± 2 ^bc^	−1.5 ± 0.2 ^cd^	11 ± 2 ^a^	13± 3 ^ab^
SA + Pe_0.30_	87.5 ± 0.8^a^	−1.80 ± 0.03 ^g^	8.9 ± 0.9 ^bc^	10 ± 1 ^c^

Values with different letters within the same column differ significantly at *p* < 0.05 levels. Samples with *a* present the highest value, while samples with *d* the lowest. L* (luminosity), a* (red/green index) and b* (yellow/blue index).

## Supplementary Materials

The following are available online at http://www.mdpi.com/1996-1944/11/10/1980/s1, Figure S1: ATR-IR spectra of SA 0.15, Pe 0.15 and SA + Pe 0.15.
