# Supplementary figures and images for: Characterization of Active Edible Films based on Citral Essential Oil, Alginate and Pectin
Source: Materials (Basel). 2018 Oct 15;11(10):1980. doi: 10.3390/ma11101980 (PMC6212942; doi:10.3390/ma11101980)

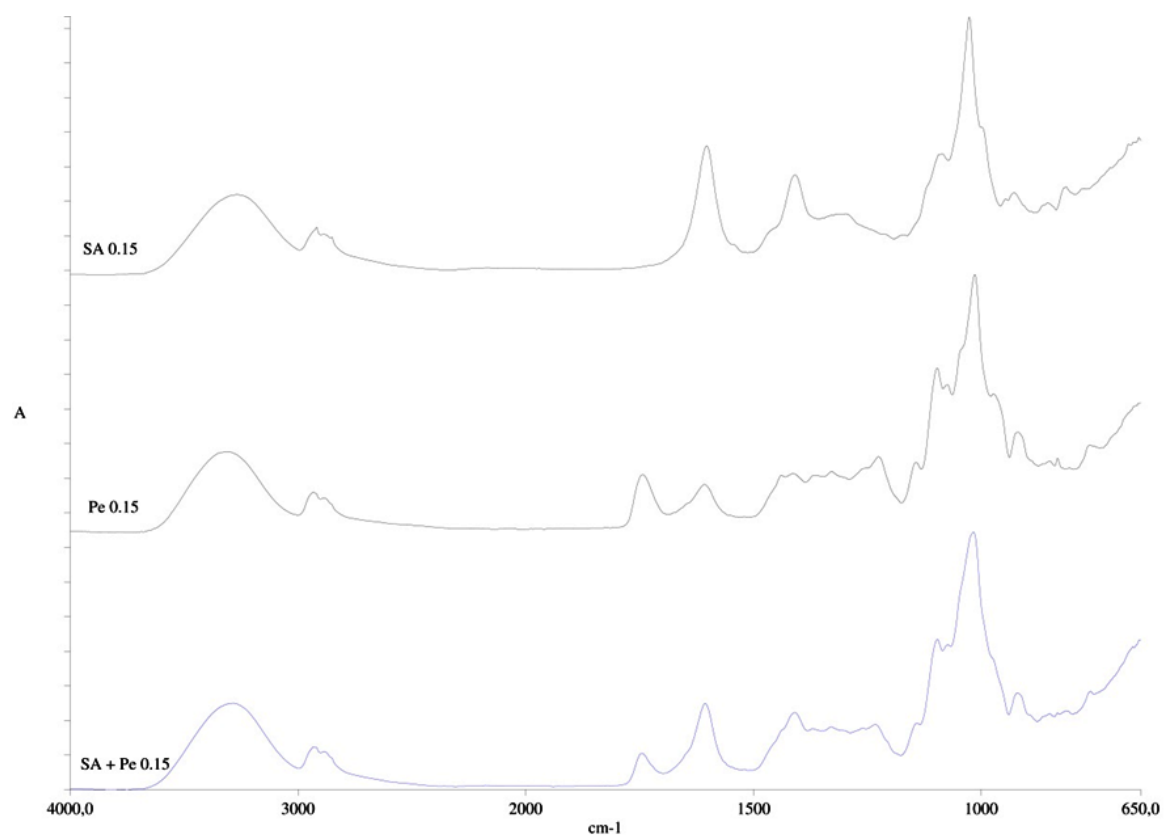

**Figure S1.** ATR-IR spectra of SA 0.15, Pe 0.15 and SA + Pe 0.15.

Supplement: Supplementary file 1 [file materials-11-01980-s001.pdf]
